# Possible severe piperacillin-tazobactam–associated drug-induced liver injury after re-exposure: a case report

**DOI:** 10.3389/fmed.2026.1977830

**Published:** 2026-10-22

**Authors:** Jingwen Ren, Weifeng Yu, Yanli Dai

**Affiliations:** Department of Pharmacy, Affiliated Meishan Hospital of Chengdu University of Traditional Chinese Medicine/Traditional Chinese Medicine Hospital of Meishan, Meishan, China

**Keywords:** adverse drug reaction, case report, drug re-exposure, drug-induced liver injury, piperacillin-tazobactam

## Abstract

**Background:**

Drug-induced liver injury (DILI) is an important cause of acute liver failure, and antimicrobial agents are among its most frequent causes. However, the association of clinically apparent liver injury with piperacillin-tazobactam is uncommon.

**Case presentation:**

A 73-year-old man with normal liver biochemistry at admission received empirical piperacillin-tazobactam for suspected pulmonary and central nervous system infection. Liver biochemical abnormalities were first detected after 4 days of treatment. The patient’s aspartate aminotransferase (AST) and alanine aminotransferase (ALT) levels markedly decreased after the withdrawal of suspected hepatotoxic drugs and supportive treatment, whereas alkaline phosphatase (ALP), gamma-glutamyl transferase (GGT), and total bilirubin levels continued to increase over the same period. Piperacillin-tazobactam was subsequently restarted because of recurrent fever and concern about inadequately controlled bacterial infection. However, liver injury worsened sharply by then, with jaundice, elevations in marked aminotransferase and alkaline phosphatase levels, and an increase in international normalized ratio (INR). After evaluation of alternative causes, DILI was considered the most likely diagnosis. Piperacillin-tazobactam was discontinued again, and supportive treatment, including double plasma molecular adsorption system therapy, was provided; AST and ALT levels and INR improved substantially thereafter, whereas ALP, GGT, and total bilirubin levels remained abnormal, indicating that cholestasis had not yet resolved at discharge.

**Conclusion:**

The temporal association, improvement after drug withdrawal, and pronounced deterioration after re-exposure support a diagnosis of possible piperacillin-tazobactam–associated DILI. Clear documentation and communication of suspected adverse drug reactions during transfers of care are essential to prevent inadvertent re-exposure.

## Introduction

Piperacillin-tazobactam is a widely used antipseudomonal penicillin and beta-lactamase inhibitor combination. Its reported adverse effects include hypersensitivity reactions, hematological abnormalities, cutaneous reactions, and neurological complications. Drug-induced liver injury (DILI) is generally caused by a medicine or its metabolites (1). Its pathogenesis is incompletely understood and may involve direct toxicity, idiosyncratic immune responses, mitochondrial dysfunction, and alterations in the gut microbiota.

The association of severe cholestatic-pattern DILI with piperacillin-tazobactam has been infrequently reported. Maheshwari et al. described a 73-year-old patient with suspected hepatocellular DILI after being administered piperacillin-tazobactam medication (2, 3). In this study, we report a patient who developed liver injury during piperacillin-tazobactam treatment, whose condition improved after drug withdrawal, and who experienced pronounced biochemical deterioration after re-exposure. This case illustrates the diagnostic value of a carefully reconstructed medication timeline and the importance of communicating suspected adverse drug reactions across clinical teams.

## Case presentation

### Patient information and clinical findings

A 73-year-old man was admitted on 10 May 2026 after 9 days of recurrent fever accompanied by altered consciousness and foaming at the mouth during febrile episodes. The patient had been administered intravenous levofloxacin lactate at another hospital from 5 May 2026 to 9 May 2026, however without clinical improvement. He had no history of hepatitis, tuberculosis, diabetes, cerebrovascular disease, allergy, alcohol use, or smoking and also had no known family history of inherited disease.

At admission at our hospital, the patient was somnolent but opened his eyes to voice. His body temperature was 39.5 °C, pulse rate was 78 beats per min, respiratory rate was 17 breaths per min, and blood pressure was 115/68 mmHg. An arterial blood gas analysis showed an oxygen saturation of 98.2%, a PaO_2_ of 101 mmHg, a PaCO_2_ of 28.2 mmHg, a pH of 7.482, a lactate level of 0.7 mmol/L, and a bicarbonate level of 21.1 mmol/L. Laboratory testing showed a white blood cell count of 7.25 × 10^9^ cells/L, comprising neutrophils (88.70%), lymphocytes (4.40%), monocytes (6.8%), eosinophils (0.00%), and basophils (0.1%); a red blood cell count of 3.68 × 10^12^ cells/L; a hemoglobin level of 119 g/L; and a platelet count of 156 × 10^9^ platelets/L. The serum creatinine level was 85 µmol/L, and the estimated glomerular filtration rate was 81.50 mL/min/1.73 m^2^. A chest CT scan showed partial consolidation in both lower lobes. The working diagnoses were pulmonary infection and central nervous system infection.

### Treatment course

Empirical intravenous piperacillin-tazobactam was started at admission. This product was manufactured by CSPC Zhongnuo Pharmaceutical (batch: 0372603114; vial strength: 2.25 g), and the administered regimen is shown in Table 1. On 12 May 2026, the cerebrospinal fluid bacterial culture was negative. A cerebrospinal fluid analysis showed a protein level of 774.51 mg/L, a positive Pandy’s test result (1+), a chloride level of 120 mmol/L, and a glucose level of 2.54 mmol/L. A contrast-enhanced brain MRI showed mild swelling of the bilateral frontal lobes, right temporal lobe, right insula, and right hippocampus; linear leptomeningeal enhancement and scattered small patchy parenchymal enhancement were also observed. Intravenous acyclovir was added because fever persisted.

**Table 1:** Medication exposure during hospitalization.

Drug	Dose and route	Treatment period
Piperacillin-tazobactam	4.5 g every 8 h, intravenous infusion	10 May 2026 to 31 May 2026; 3 June 2026 to 8 June 2026
Acyclovir	0.5 g every 8 h, intravenous infusion	12 May 2026 to 31 May 2026
Glutathione	1.2 g once daily, intravenous infusion	14 May 2026 to 23 May 2026
Polyene phosphatidylcholine	232.5 mg once daily, intravenous infusion	14 May 2026 to 15 June 2026
Human albumin	10 g once daily, intravenous infusion	14 May 2026 to 18 May 2026; 4 June 2026 to 10 June 2026
Magnesium isoglycyrrhizinate	200 mg once daily, intravenous infusion	31 May 2026 to 15 June 2026

Abnormal liver biochemistry was first detected after 4 days of piperacillin-tazobactam treatment. Both infection-associated liver injury and DILI were considered. Glutathione and polyene phosphatidylcholine were started, and human albumin was administered for hypoalbuminemia. On 31 May 2026, cutaneous and scleral jaundice developed, which were accompanied by abdominal tenderness. An abdominal CT scan showed no biliary obstruction. The test results for schistosomiasis, hepatitis A virus IgM, hepatitis B surface antigen, hepatitis C virus antibody, hepatitis E virus IgM, Epstein–Barr virus IgM, cytomegalovirus IgM, antinuclear antibody, antimitochondrial M2 antibody, anti–smooth muscle antibody, anti–liver–kidney microsomal type 1 antibody, and anti–liver cytosol type 1 antibody were negative. Acute liver injury was added to the diagnoses. On the recommendation of the clinical pharmacist, piperacillin-tazobactam and acyclovir were stopped, and supportive pharmacotherapy with polyene phosphatidylcholine and magnesium isoglycyrrhizinate was continued.

The patient was transferred to the gastroenterology department on 2 June 2026 because of persistent liver biochemical abnormalities. On 3 June 2026, fever recurred to 39 °C with cough and sputum production; the procalcitonin level was 0.3 ng/mL. Piperacillin-tazobactam was restarted because clinicians suspected an inadequately controlled pulmonary bacterial infection after antimicrobial withdrawal. By 6 June 2026, aminotransferase (ALT) concentrations had increased to more than 10 times its upper limit of normal (ULN), and marked jaundice was present. Double plasma molecular adsorption system (DPMAS) therapy was initiated. Liver biochemistry worsened sharply on 7 June 2026. After causality assessment by the clinical pharmacist, piperacillin-tazobactam was discontinued again. The last day of administration was 8 June 2026, and the drug was not used thereafter. Additional DPMAS sessions were performed on 8, 10, 11, and 13 June 2026. The treatment timeline is shown in Figure 1.

**Figure 1:**
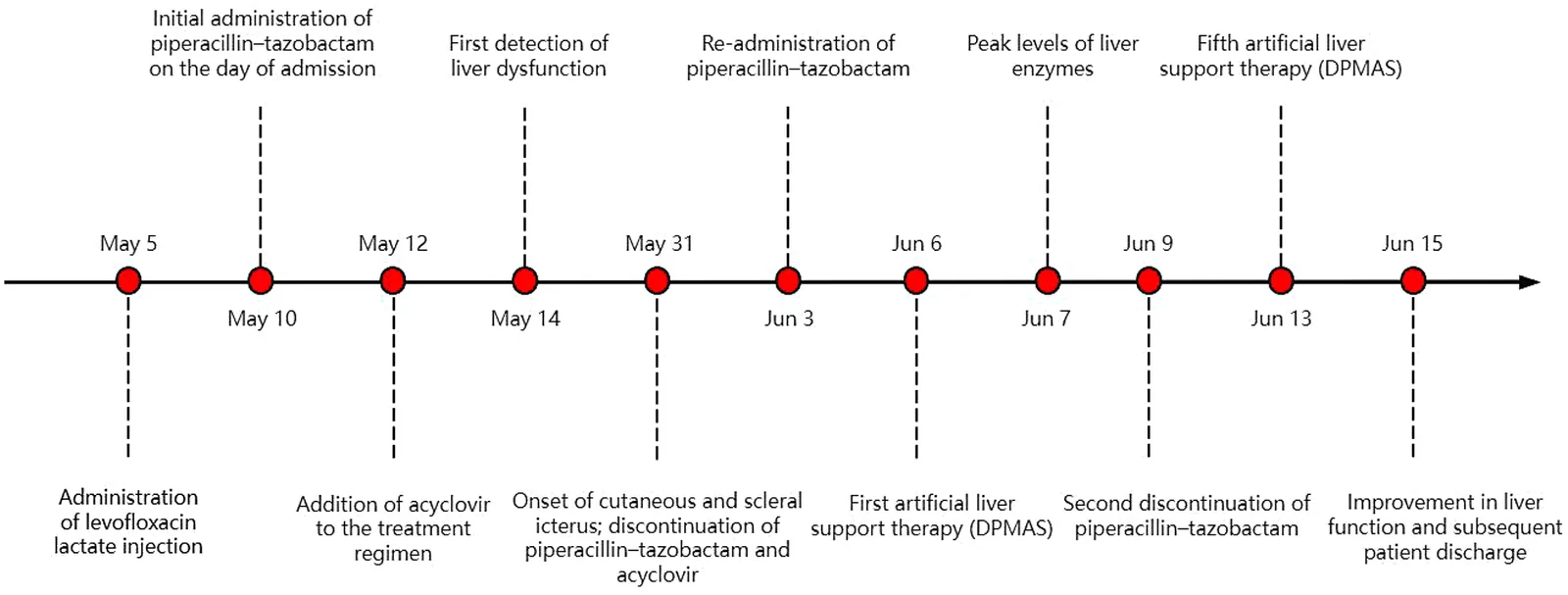
The timeline of medication exposure, liver injury, drug withdrawal, re-exposure, and DPMAS treatment.

### Diagnostic assessment

Acute DILI is generally identified when at least one of the following biochemical thresholds is met: an alanine aminotransferase level at least five times its ULN, an alkaline phosphatase (ALP) at least two times its ULN, or an ALT level at least three times its ULN along with a total bilirubin level at least two times its ULN (4, 5). The patient subsequently developed jaundice and had elevated marked liver enzyme levels and an international normalized ratio (INR) of up to 1.97; the source guideline classified this presentation as grade 3 (severe) acute DILI (1).

The R value was calculated as (ALT/ALT ULN) divided by (ALP/ALP ULN). Using the first liver biochemistry measurements meeting the predefined DILI diagnostic thresholds on 31 May 2026, the R value was 1.61, which was interpreted as a cholestatic biochemical pattern (1). At the peak of the second episode on 7 June 2026, the R value rose to 5.23, indicating a shift to the hepatocellular pattern.

Nevertheless, DILI remains highly difficult to diagnose because no specific diagnostic biomarker is available (5). Liver biochemistry was normal at admission and became abnormal 4 days after the piperacillin-tazobactam was started; DILI was first suspected on 31 May 2026. Aminotransferase concentrations decreased after the suspected hepatotoxic drugs were stopped that same day, whereas ALP, GGT, and TBil levels continued to increase over the same period. After piperacillin-tazobactam was restarted on 3 June 2026, aspartate aminotransferase (AST) and ALT levels markedly increased and peaked on 7 and 8 June 2026, respectively (Table 2, Figure 2). No further doses were given after 8 June 2026; AST and ALT levels and INR subsequently improved, whereas ALP, GGT, and TBil levels remained abnormal and fluctuated (Figure 2). The patient had no history of hepatitis, alcohol use, or hepatobiliary disease. Testing excluded schistosomiasis, autoimmune liver disease, and biliary obstruction; serological test results for viral hepatitis (anti-HAV IgM, HBsAg, anti-HCV, and anti-HEV IgM) were negative, although serological tests for anti-HBc IgM, HBV DNA, HCV RNA, anti-HEV IgG, and HEV RNA were not performed. Cerebrospinal fluid cultures on 12, 16, and 29 May 2026 and sputum culture on 13 May 2026 were negative. The procalcitonin level was 0.3 ng/mL on 3 June 2026 (reference range, 0–0.077 ng/mL), and the Sequential Organ Failure Assessment score was less than 2. Although sepsis was considered unlikely, infection-associated liver injury could not be excluded completely.

**Table 2:** Serial laboratory measurements during hospitalization.

Date	ALT/AST, U/L (9–50/15–40)	ALP, U/L (45–125)	GGT, U/L (15–73)	TBil/DBil, μmol/L (0–21/0–6.8)	Albumin, g/L (35–50)	INR	Creatinine, μmol/L (53–123)	WBC, ×10^9^ cells/L (3.5–9.5)	*R*
10 May 2026	39.9/45.6	61.15	28.86	11.9/3.72	30.0	1.16	85	7.25	1.63
14 May 2026	151.1/176.9	143.31	133.54	10.15/4.26	24.7	1.22	81	6.2	2.64
19 May 2026	78.9/60.5	160.3	141.5	15.6/4.2	40.3	1.19	NA	5.72	1.23
31 May 2026	410.7/502.3	638.21	566.36	141.13/92.77	29.3	1.17	63	5.12	1.61
1 June 2026	329.1/237.2	684.8	619.8	155.7/99.1	29.3	1.12	69.3	4.03	1.20
2 June 2026	237.5/136.5	885.43	762.7	189.99/131.73	30.1	NA	64	5.28	0.67
4 June 2026	200.1/156.8	858.8	775.0	326.9/206.4	28.5	1.21	NA	7.0	0.58
5 June 2026	259.8/265.2	702.8	662.2	308.1/191.2	28.6	1.22	NA	NA	0.92
6 June 2026	654.2/894.2	781.22	725.89	377.0/246.8	31.1	NA	NA	NA	2.09
7 June 2026	1,263.9/1,746.1	604.64	592.73	370.03/239.01	47.6	1.95	NA	NA	5.23
8 June 2026	1,310.2/1,263.3	473.9	501.4	410.8/254.9	27.8	1.97	NA	NA	6.91
9 June 2026	699.5/489.1	263.7	251.0	345.2/197.7	27.3	1.63	NA	5.29	6.63
10 June 2026	481.4/143.2	257.69	271.16	353.63/221.16	29.8	NA	NA	NA	4.67
11 June 2026	224.5/42.9	190.3	235.0	286.2/167.2	30.9	1.35	NA	NA	2.95
12 June 2026	186.5/32.1	221.8	320.2	319.0/187.6	29.0	NA	NA	5.43	2.10
13 June 2026	155.1/33.0	247.3	395.54	336.73/209.25	26.5	1.25	NA	NA	1.57
14 June 2026	88.5/26.4	190.78	274.37	274.29/171.23	26.5	1.25	NA	NA	1.16
15 June 2026	88.4/26.7	234.6	311.1	352.9/209.1	26.3	1.29	NA	NA	0.94

ALT, alanine aminotransferase; AST, aspartate aminotransferase; ALP, alkaline phosphatase; GGT, gamma-glutamyl transferase; TBil, total bilirubin; DBil, direct bilirubin; INR, international normalized ratio; WBC, white blood cell count; NA, not available.

**Figure 2:**
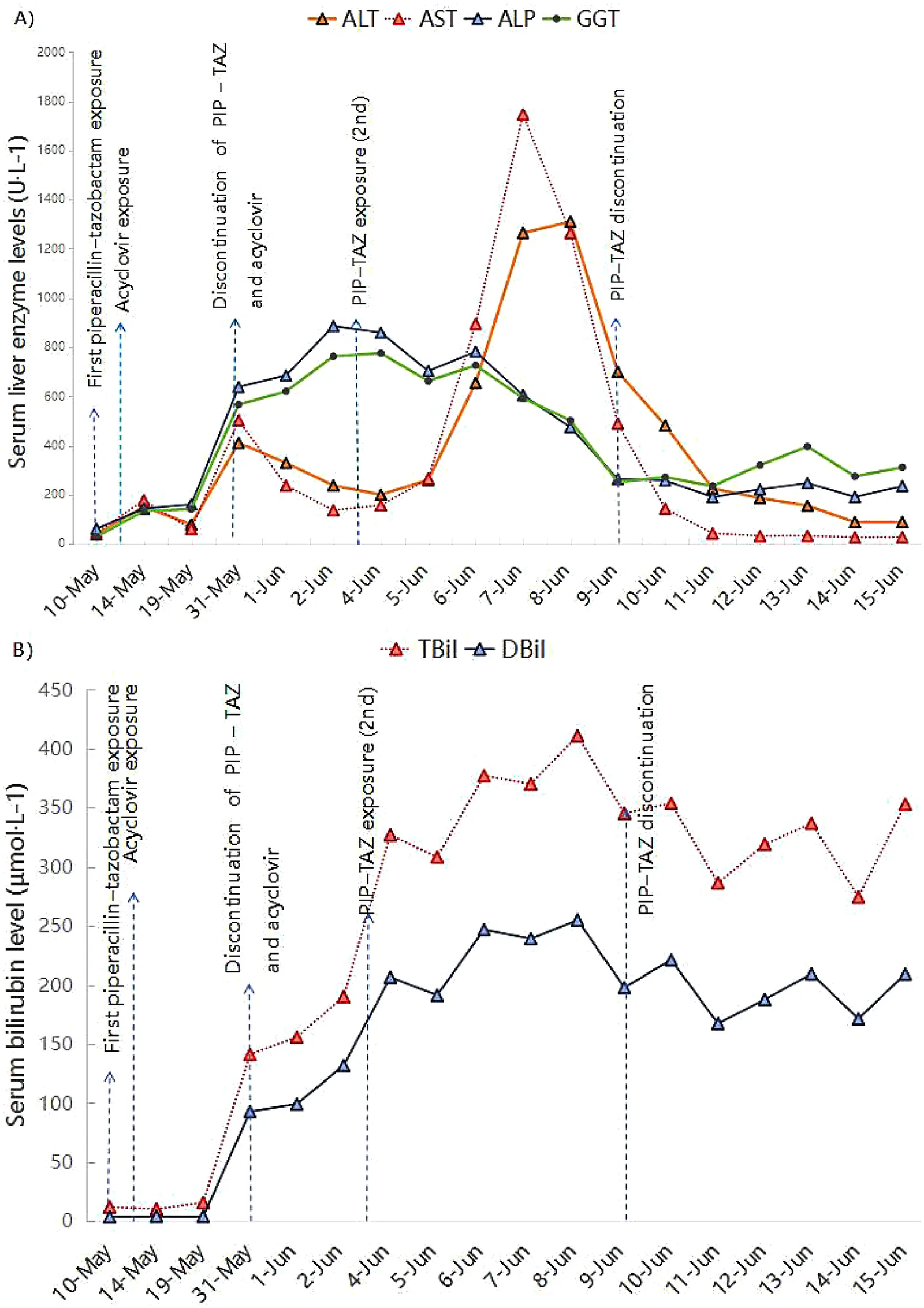
Serial liver biochemistry during hospitalization. **(A)** Aminotransferases, alkaline phosphatase, and gamma-glutamyl transferase levels. **(B)** Total and direct bilirubin levels.

Levofloxacin had been administered before admission, but liver biochemistry was normal at admission, and therefore, the drug was not restarted. At the onset of liver injury, the patient was receiving both piperacillin-tazobactam and acyclovir medications; when both were withdrawn, the liver biochemistry improved. During the severe recurrence, piperacillin-tazobactam was the only one of these agents that was reintroduced, making other drugs less likely causes. The Roussel Uclaf Causality Assessment Method (RUCAM) score was 3, indicating a possible association between piperacillin-tazobactam and acute liver injury. The Naranjo score was 7, indicating a probable drug-related reaction and supporting the RUCAM assessment of drug causality (6).

### Therapeutic intervention

When abnormal liver biochemistry was first detected on 14 May 2026, glutathione and polyene phosphatidylcholine were administered. After the marked deterioration on 31 May 2026, treatment was changed to polyene phosphatidylcholine plus magnesium isoglycyrrhizinate. Five DPMAS sessions were performed in addition to the pharmacological interventions listed in Table 1. AST and ALT levels improved during treatment, but the patient was discharged on 15 June 2026 with persistent hyperbilirubinemia and cholestatic biochemical abnormalities.

### Follow-up and outcomes

At discharge, AST levels had normalized, ALT levels had decreased to 88.4 U/L, and the INR had decreased to 1.29, whereas ALP, GGT, and total bilirubin levels remained markedly abnormal. No subsequent laboratory follow-up was available when the manuscript of this study was completed, and therefore, the final clinical and biochemical outcomes remain unknown.

## Discussion

DILI is an important cause of acute liver failure, and antimicrobial agents are among its most frequent triggers (1, 7). Piperacillin-tazobactam is widely used, but its reported DILI frequency among hospitalized patients is approximately 0.097% (8). The large number of exposed patients makes early recognition clinically important despite the low reported frequency. Published reports describe predominantly cholestatic or mixed biochemical patterns, often with a latency of approximately 2 weeks (9, 10).

The present case shares features with the report by Maheshwari and colleagues (2) but differs in biochemical phenotype. In that report, hepatocellular-pattern DILI developed after a 2-week treatment course, with an aspartate aminotransferase level of 1,248 U/L, an ALT level of 272 U/L, and an ALP level of 220 U/L, followed by gradual recovery after drug withdrawal. In our patient, the injury was characterized by rapid reversibility of the hepatocellular component but persistent cholestasis. After the first withdrawal, AST and ALT levels decreased, whereas ALP, GGT, and total bilirubin levels continued to increase. After the second withdrawal, AST and ALT levels and INR decreased markedly, whereas ALP, GGT, and TBil levels remained abnormal. The same pattern was observed after both withdrawals, suggesting that the hepatocellular and cholestatic components of liver injury in this case differed in reversibility. This difference in injury pattern suggests that the pathophysiological mechanisms of liver injury may be heterogeneous between individuals even for the same drug.

The initial decrease in the levels of aminotransferases after the withdrawal of piperacillin-tazobactam medication supported a positive dechallenge. After transfer to another clinical service, the drug was restarted and liver injury worsened sharply. Re-exposure to a suspected causative drug should generally be avoided unless the potential benefit is substantial and no suitable alternative exists (1). In this case, the sequence raises concern that information on the suspected adverse drug reaction (ADR) was not communicated effectively during transfer. Medication reconciliation, visible adverse-reaction documentation, and explicit handover may reduce the risk of inadvertent re-exposure.

The clinical pharmacist contributed to reconstructing the exposure timeline, assessing causality, and recommending drug withdrawal. Therefore, this case also illustrates how pharmacist involvement can support the recognition and prevention of serious medication-related harm.

## Conclusion

To conclude, in the case of the patient in this study, hepatocellular injury and coagulation reduced substantially after withdrawal of the suspected drug, whereas cholestatic abnormalities remained substantial at discharge; because no postdischarge laboratory follow-up was available, the subsequent duration and completeness of biochemical recovery were unknown. With the widespread clinical use of piperacillin-tazobactam, its safety data still need to be accumulated. Patients receiving piperacillin-tazobactam should undergo clinically appropriate liver biochemical monitoring when symptoms or risk factors raise concern. Adverse drug reaction surveillance should be continued, with emphasis on new and serious reactions and strengthened vigilance for high-risk drug categories such as anti-infective agents. In addition, during transfers of care, clinicians should properly document and communicate suspected ADRs to prevent the serious consequences of inadvertent re-exposure.

## Data Availability

The original contributions presented in the study are included in the article/Supplementary Material, and further inquiries can be directed to the corresponding author.

## References

[1] Technology Committee on DILI Prevention and Management, Chinese Medical Biotechnology Association, Study Group of Drug-Induced Liver Disease, Chinese Medical Association for the Study of Liver Diseases. Chinese guideline for diagnosis and management of drug-induced liver injury (2023 version). Zhonghua Gan Zang Bing Za Zhi. (2023) 31(4):355–84. 10.3760/cma.j.cn501113-20230419-00176-1 37248976 PMC12814733

[2] Maheshwari PK Fahim P Yoo J Harrison A. S4482 Piperacillin-tazobactam and hepatic complications: a case report on drug-induced liver injury. Am J Gastroenterol. (2024) 119(10S):S2862. 10.14309/01.ajg.0001047296.16554.19

[3] Maheshwari PK Maddox A Yoo J Harrison A Fahim P. From therapy to toxicity: a rare case of acute piperacillin-tazobactam-associated drug-induced liver injury. Cureus. (2025) 17(9):e93434. 10.7759/cureus.93434 41170219 PMC12568140

[4] Council for International Organizations of Medical Sciences. Drug-Induced Liver Injury (DILI): Current Status and Future Directions for Drug Development and the Post-Market Setting. Geneva: CIOMS (2020).

[5] Andrade RJ Aithal GP Björnsson ES Kaplowitz N Kullak-Ublick GA Larrey D. EASL Clinical Practice Guidelines: drug-induced liver injury. J Hepatol. (2019) 70(6):1222–61. 10.1016/j.jhep.2019.02.014 30926241

[6] Naranjo CA Busto U Sellers EM Sandor P Ruiz I Roberts EA. A method for estimating the probability of adverse drug reactions. Clin Pharmacol Ther. (1981) 30(2):239–45. 10.1038/clpt.1981.154 7249508

[7] Hosack T Damry D Biswas S. Drug-induced liver injury: a comprehensive review. Therap Adv Gastroenterol. (2023) 16:17562848231163410. 10.1177/17562848231163410 PMC10031606 36968618

[8] Bjornsson RA Sigurdsson SS Arnarson DT Logason E Bjornsson ES. The frequency of drug-induced liver injury due to antibiotics among hospitalised patients. Drug Saf. (2025) 48:795–804. 10.1007/s40264-025-01541-w 40072769

[9] National Institute of Diabetes and Digestive and Kidney Diseases. Piperacillin-tazobactam. LiverTox: clinical and research information on drug-induced liver injury (2020). Available online at: https://www.ncbi.nlm.nih.gov/books/NBK548825/ (Accessed July 10, 2026).

[10] Wang J Wang S Wu C Deng Z. Antibiotic-associated vanishing bile duct syndrome: a real-world retrospective and pharmacovigilance database analysis. Infection. (2024) 52:891–9. 10.1007/s15010-023-02132-6 37996645

