# Supplementary material for: Possible severe piperacillin-tazobactam–associated drug-induced liver injury after re-exposure: a case report
Source: Front Med (Lausanne). 2026 Oct 22;13:1977830. doi: 10.3389/fmed.2026.1977830 (PMC13735729; doi:10.3389/fmed.2026.1977830)
Supplement: Supplementary file 1 [file Datasheet1.docx]

**Naranjo Adverse Drug Reaction Probability Scale**

| **Question** | **Yes** | **No** | **Do not know** | **Score** | **Evidence from this case** |
| --- | --- | --- | --- | --- | --- |
| 1. Are there previous conclusive reports on this reaction? | ☑ +1 | 0 | 0 | **+1** | Hepatotoxicity is labelled in the product characteristics of piperacillin-tazobactam and has been described in published reports. |
| 2. Did the adverse event appear after the suspected drug was administered? | ☑ +2 | −1 | 0 | **+2** | Liver biochemistry was normal at admission; aminotransferase elevations were first detected on day 4 (May 14), and the liver biochemistry first met the diagnostic threshold for acute DILI on May 31. |
| 3. Did the adverse reaction improve when the drug was discontinued or a specific antagonist was administered? | ☑ +1 | 0 | 0 | **+1** | Aminotransferases declined after piperacillin-tazobactam and acyclovir were withdrawn on May 31 (positive dechallenge). |
| 4. Did the adverse event reappear when the drug was re-administered? | ☑ +2 | −1 | 0 | **+2** | Piperacillin-tazobactam was readministered as anti-infective therapy on June 3, and AST and ALT peaked on June 7 and June 8, respectively. |
| 5. Are there alternative causes (other than the drug) that could on their own have caused the reaction? | −1 | +2 | ☑ 0 | **0** | Serological tests for HAV (anti-HAV IgM), HBV (HBsAg), HCV (anti-HCV), and HEV (anti-HEV IgM) were negative; however, anti-HBc IgM, HBV DNA, HCV RNA, anti-HEV IgG, and HEV RNA were not performed, so viral hepatitis could not be completely excluded. Autoimmune markers were negative, schistosomiasis was excluded, imaging showed no biliary obstruction, and there was no history of alcohol use or recent hypotension. This item was therefore scored 0 (do not know). |
| 6. Did the reaction reappear when a placebo was given? | −1 | +1 | ☑ 0 | **0** | A placebo challenge was not performed. |
| 7. Was the drug detected in blood (or other fluids) in concentrations known to be toxic? | +1 | 0 | ☑ 0 | **0** | Serum drug concentrations were not measured. |
| 8. Was the reaction more severe when the dose was increased or less severe when the dose was decreased? | +1 | 0 | ☑ 0 | **0** | The dose of piperacillin-tazobactam was not changed during hospitalisation. |
| 9. Did the patient have a similar reaction to the same or similar drugs in any previous exposure? | +1 | ☑ 0 | 0 | **0** | The patient had no known previous exposure to piperacillin-tazobactam or related antibiotics. |
| 10. Was the adverse event confirmed by any objective evidence? | ☑ +1 | 0 | 0 | **+1** | The reaction was documented by serial liver biochemistry and coagulation testing, together with abdominal imaging. |
| **TOTAL SCORE** | | | | 7 | **Causal relationship: probable (total score 5–8)** |

**Interpretation of the total score:**

greater than 8: the adverse drug reaction is “definite”;

5–8: the adverse drug reaction is “probable”;

1–4: the adverse drug reaction is “possible”;

0 or less: the adverse drug reaction is “doubtful”.
