# Supplementary material for: Possible severe piperacillin-tazobactam–associated drug-induced liver injury after re-exposure: a case report
Source: Front Med (Lausanne). 2026 Oct 22;13:1977830. doi: 10.3389/fmed.2026.1977830 (PMC13735729; doi:10.3389/fmed.2026.1977830)
Supplement: Supplementary file 2 [file Datasheet2.docx]

**RUCAM worksheet for cholestatic and mixed liver injury**

| **Item (cholestatic or mixed liver injury)** | **Score** | **Result** | **Details** |
| --- | --- | --- | --- |
| **1. Time to onset from the beginning of the drug**  • 5–90 days (rechallenge: 1–90 days)  • <5 or >90 days (rechallenge: >90 days)  • Alternative: time to onset from cessation of the drug: ≤30 days (except for slowly metabolized chemicals: >30 days) | +2  +1  +1 | ☑  □  □ | The onset of the DILI event was defined as May 31, when ALT first exceeded 5×ULN. The corresponding latency was 21 days from the initiation of piperacillin-tazobactam (May 10 to May 31). |
| **2. Course of ALP after cessation of the drug**  • Decrease ≥50% within 180 days  • Decrease <50% within 180 days  • No information, persistence, increase, or continued drug use | +2  +1  0 | ☑  □  □ | Following the final administration of piperacillin-tazobactam on June 8, when ALP was 473.9 U/L, ALP decreased to 190.3 U/L by June 11—a reduction of approximately 60% within three days, fulfilling the criterion of a ≥50% decrease within 180 days. |
| **3. Risk factors**  • Alcohol use (current drinks/d: >2 for women, >3 for men)  • Alcohol use (current drinks/d: ≤2 for women, ≤3 for men)  • Pregnancy  • Age ≥55 years  • Age <55 years | 0  +1  0  +1  0 | □  □  □  ☑  □ | The patient was a 73-year-old man (age ≥55 years) with no history of alcohol consumption. |
| **4. Concomitant use of drug(s)/herb(s)**  • None or no information  • Concomitant drug/herb with incompatible time to onset  • Concomitant drug/herb with time to onset 5–90 days  • Concomitant drug/herb known as hepatotoxin and with time to onset 5–90 days  • Concomitant drug/herb with evidence for its role in this case (positive rechallenge or validated test) | 0  0  −1  −2  −3 | □  □  □  ☑  □ | Acyclovir was given concomitantly with piperacillin-tazobactam during the first episode of liver injury. Hepatotoxicity is mentioned in the package insert of acyclovir. Acyclovir was administered from May 12 to May 31, and the latency to the qualifying onset (May 31) was 19 days, which fell within the 5–90 day window and was therefore temporally compatible. |
| **5. Search for alternative causes**  **Group I (7 causes)**  • HAV: anti-HAV IgM  • HBV: HBsAg, anti-HBc IgM, HBV DNA  • HCV: anti-HCV, HCV RNA  • HEV: anti-HEV IgM, anti-HEV IgG, HEV RNA  • Hepatobiliary sonography / Doppler / CT / MRC  • Alcoholism (AST/ALT ≥2)  • Acute recent hypotension history (particularly if underlying heart disease)  **Group II (5 causes)**  • Complications of underlying disease(s) such as sepsis, metastatic malignancy, autoimmune hepatitis, chronic hepatitis B or C, primary biliary cholangitis or sclerosing cholangitis, genetic liver diseases  • Infection suggested by PCR and titer change for CMV (anti-CMV IgM, anti-CMV IgG)  • EBV (anti-EBV IgM, anti-EBV IgG)  • HSV (anti-HSV IgM, anti-HSV IgG)  • VZV (anti-VZV IgM, anti-VZV IgG)  **Evaluation of Groups I and II**  • All causes – Groups I and II – reasonably ruled out  • The 7 causes of Group I ruled out  • 6 or 5 causes of Group I ruled out  • Less than 5 causes of Group I ruled out  • Alternative cause highly probable | +2  +1  0  −2  −3 | ☑  □  □  □  ☑  ☑  ☑  ☑  ☑  ☑  □  □  □  □  □  ☑  □ | Serological tests for hepatitis A (anti-HAV IgM), hepatitis B (HBsAg), hepatitis C (anti-HCV), and hepatitis E (anti-HEV IgM) were negative, and whole-abdominal CT showed no biliary obstruction; the patient had no history of alcohol consumption or recent hypotension. However, anti-HBc IgM, HBV DNA, HCV RNA, anti-HEV IgG, and HEV RNA were not performed, so hepatitis B, hepatitis C, and hepatitis E could not be regarded as fully excluded. Only four of the seven Group I causes were therefore ruled out.  Antinuclear antibody, anti-mitochondrial M2 antibody, anti-smooth muscle antibody, anti-LKM-1, anti-LC-1, EBV IgM, and CMV IgM were negative; HSV and VZV serologies were not performed, and Group II causes could not be completely excluded. This item was therefore scored −2 (less than 5 causes of Group I ruled out). |
| **6. Previous hepatotoxicity of the drug**  • Reaction labelled in the product characteristics  • Reaction published but unlabelled  • Reaction unknown | +2  +1  0 | ☑  □  □ | Hepatotoxicity is labelled in the product characteristics of piperacillin-tazobactam. |
| **7. Response to unintentional re-exposure**  • Doubling of ALP with the drug alone, provided ALP was below 2 × ULN before re-exposure  • Doubling of ALP with the drug(s)/herb(s) already given at the time of the first reaction  • Increase of ALP but less than 1 × ULN under the same conditions as the first administration  • Other situations | +3  +1  −2  0 | □  □  □  ☑ | The ALP immediately before re-exposure (June 2) was 885.43 U/L (approximately 7.1×ULN); after piperacillin-tazobactam was readministered on June 3, the ALP on June 6 was 781.22 U/L—no doubling occurred, and the value was in fact lower than before re-exposure. |
| **Total score** | 3 |  | **Causal relationship: possible** |

**Interpretation of the total score:**

0 or less: the liver injury is unrelated to the suspected product;

1–2: the causal relationship is “unlikely”;

3–5: the causal relationship is “possible”;

6–8: the causal relationship is “probable”;

greater than 8: the causal relationship is “highly probable”.

**Naranjo Adverse Drug Reaction Probability Scale**

| **Question** | **Yes** | **No** | **Do not know** | **Score** | **Evidence from this case** |
| --- | --- | --- | --- | --- | --- |
| 1. Are there previous conclusive reports on this reaction? | ☑ +1 | 0 | 0 | **+1** | Hepatotoxicity is labelled in the product characteristics of piperacillin-tazobactam and has been described in published reports. |
| 2. Did the adverse event appear after the suspected drug was administered? | ☑ +2 | −1 | 0 | **+2** | Liver biochemistry was normal at admission; aminotransferase elevations were first detected on day 4 (May 14), and the liver biochemistry first met the diagnostic threshold for acute DILI on May 31. |
| 3. Did the adverse reaction improve when the drug was discontinued or a specific antagonist was administered? | ☑ +1 | 0 | 0 | **+1** | Aminotransferases declined after piperacillin-tazobactam and acyclovir were withdrawn on May 31 (positive dechallenge). |
| 4. Did the adverse event reappear when the drug was re-administered? | ☑ +2 | −1 | 0 | **+2** | Piperacillin-tazobactam was readministered as anti-infective therapy on June 3, and AST and ALT peaked on June 7 and June 8, respectively. |
| 5. Are there alternative causes (other than the drug) that could on their own have caused the reaction? | −1 | +2 | ☑ 0 | **0** | Serological tests for HAV (anti-HAV IgM), HBV (HBsAg), HCV (anti-HCV), and HEV (anti-HEV IgM) were negative; however, anti-HBc IgM, HBV DNA, HCV RNA, anti-HEV IgG, and HEV RNA were not performed, so viral hepatitis could not be completely excluded. Autoimmune markers were negative, schistosomiasis was excluded, imaging showed no biliary obstruction, and there was no history of alcohol use or recent hypotension. This item was therefore scored 0 (do not know). |
| 6. Did the reaction reappear when a placebo was given? | −1 | +1 | ☑ 0 | **0** | A placebo challenge was not performed. |
| 7. Was the drug detected in blood (or other fluids) in concentrations known to be toxic? | +1 | 0 | ☑ 0 | **0** | Serum drug concentrations were not measured. |
| 8. Was the reaction more severe when the dose was increased or less severe when the dose was decreased? | +1 | 0 | ☑ 0 | **0** | The dose of piperacillin-tazobactam was not changed during hospitalisation. |
| 9. Did the patient have a similar reaction to the same or similar drugs in any previous exposure? | +1 | ☑ 0 | 0 | **0** | The patient had no known previous exposure to piperacillin-tazobactam or related antibiotics. |
| 10. Was the adverse event confirmed by any objective evidence? | ☑ +1 | 0 | 0 | **+1** | The reaction was documented by serial liver biochemistry and coagulation testing, together with abdominal imaging. |
| **TOTAL SCORE** | | | | 7 | **Causal relationship: probable (total score 5–8)** |

**Interpretation of the total score:**

greater than 8: the adverse drug reaction is “definite”;

5–8: the adverse drug reaction is “probable”;

1–4: the adverse drug reaction is “possible”;

0 or less: the adverse drug reaction is “doubtful”.
